# An Analysis of the Anti-Neuropathic Effects of Qi She Pill Based on Network Pharmacology

**DOI:** 10.1155/2020/7193832

**Published:** 2020-05-04

**Authors:** Yong-jia Song, Jia-min Bao, Long-yun Zhou, Gan Li, Kim Sia Sng, Yong-jun Wang, Qi Shi, Xue-jun Cui

**Affiliations:** ^1^Spine Disease Institute, Longhua Hospital, Shanghai University of Traditional Chinese Medicine, Shanghai 200032, China; ^2^Key Laboratory of Theory and Therapy of Muscles and Bones, Ministry of Education, Shanghai University of Traditional Chinese Medicine, Shanghai 200032, China; ^3^Rehabilitation Medicine College, Shanghai University of Traditional Chinese Medicine, Shanghai 200000, China; ^4^Shanghai University of Traditional Chinese Medicine, Shanghai 200000, China

## Abstract

**Background:**

Qi She Pill (QSP) is a traditional prescription for the treatment of neuropathic pain (NP) that is widely used in China. However, no network pharmacology studies of QSP in the treatment of NP have been conducted to date.

**Objective:**

To verify the potential pharmacological effects of QSP on NP, its components were analyzed via target docking and network analysis, and network pharmacology methods were used to study the interactions of its components.

**Materials and Methods:**

Information on pharmaceutically active compounds in QSP and gene information related to NP were obtained from public databases, and a compound-target network and protein-protein interaction network were constructed to study the mechanism of action of QSP in the treatment of NP. The mechanism of action of QSP in the treatment of NP was analyzed via Gene Ontology (GO) biological process annotation and Kyoto Gene and Genomics Encyclopedia (KEGG) pathway enrichment, and the drug-like component-target-pathway network was constructed.

**Results:**

The compound-target network contained 60 compounds and 444 corresponding targets. The key active compounds included quercetin and beta-sitosterol. Key targets included PTGS2 and PTGS1. The protein-protein interaction network of the active ingredients of QSP in the treatment of NP featured 48 proteins, including DRD2, CHRM, *β*2-adrenergic receptor, HTR2A, and calcitonin gene-related peptide. In total, 53 GO entries, including 35 biological process items, 7 molecular function items, and 11 cell related items, were identified. In addition, eight relevant (KEGG) pathways were identified, including calcium, neuroactive ligand-receptor interaction, and cAMP signaling pathways.

**Conclusion:**

Network pharmacology can help clarify the role and mechanism of QSP in the treatment of NP and provide a foundation for further research.

## 1. Introduction

Neuropathic pain (NP) is a common condition globally [1]. With a complex pathogenesis, reliable treatments are currently unavailable; consequently, NP has a substantial negative impact on quality of life, and it causes a heavy social burden. NP was defined by the International Pain Society in 2011 as “damaged by the somatosensory nervous system or pain caused by disease” [2]. The clinical symptoms of NP mainly include hyperalgesia, allodynia, and spontaneous pain [3]. NP has a high incidence, accounting for approximately one-fifth of all cases of chronic pain, thereby causing significant damage to patients' physical and mental health [4]. In addition, NP remains a major focus of both clinical medicine and basic research.

Qi She Pill (QSP) has been approved by the China Food and Drug Administration (approval number: Z20090978), and it is widely available in China [5]. QSP is composed of six Chinese herbs, namely Caulis Sinomenii (CS, Qing Feng Teng), Calculus Bovis (BC, Niu Huang), Chuanxiong Rhizoma (CR, Chuan Xiong), Stephaniae Tetrandrae Radix (STR, Fang Ji), Hedysarum Multijugum Maxim (HMM, Huang Qi), and Moschus (MO, She Xiang). QSP has several effects, such as improving Qi, dissipating phlegm, eliminating stagnation, and relieving pain [6].

Preclinical pharmacological studies reported that QSP has analgesic effects and obvious anti-inflammatory effects against acute and chronic inflammation [7]. Pathology studies found that QSP can suppress NP. Furthermore, clinical trials proved that QSP effectively relieves pain and improves clinical symptoms and that the therapy is safe for treating NP. Phase II and III clinical trials of QSP also verified its clinical effect in patients with NP, and no obvious adverse effects were identified. QSP is widely used to treat NP in China.

The development of network pharmacology [8] has been bolstered by the rapid development of multidisciplinary fields such as pharmacy, computer science, biology, and systems biology [9]. The comprehensive observation of drugs using network pharmacology systems is achieved through network analysis covering a wide range of diseases, genes, targets, and drug networks. The results of this analysis illustrate that a disease can be regulated by a variety of different proteins/genes, and some proteins can also be regulated by a variety of diseases. Such results fully reflect the theory of traditional Chinese medicine (TCM) that the same disease can be treated with different methods and that different diseases can be treated with the same methods. In-depth studies of TCM compounds revealed the material basis and molecular mechanism of Chinese medicine pharmacodynamics [10].

**Figure 1:**
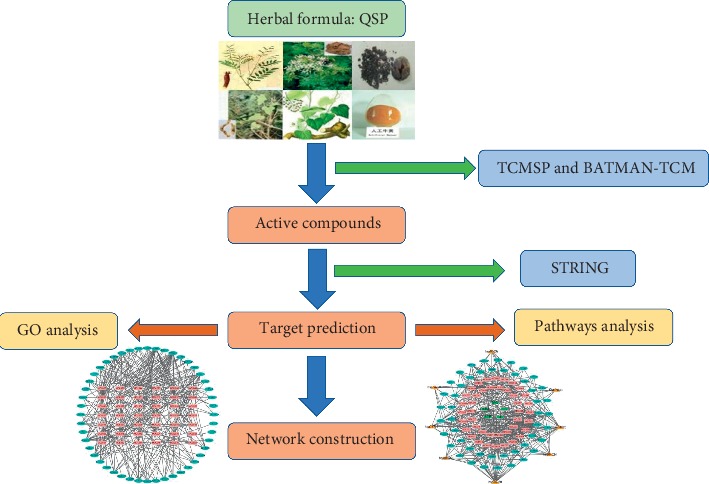
The workflow of network pharmacology analysis.

Although the analgesic effects and chemical profiling of QSP have been reported, the active compounds in QSP and the specific molecular mechanisms of QSP in treating NP are unclear, and network pharmacology-based prediction of the bioactive components and their target pathways has not been performed. Therefore, the present study explored the pharmacological mechanisms by which QSP alleviates NP and investigated the relationships among herbs, compounds, and target genes by employing QSP-based network pharmacology. First, we collected information on the bioactive compounds in QSP and retrieved candidate target genes from public databases. Network analysis was then performed to connect compounds in QSP with their target genes and related signaling pathways. Finally, based on the results of network analysis, multicompound, multitarget, and multipathway mechanisms related to QSP were predicted. The workflow is shown in Figure 1.

## 2. Methods

### 2.1. Collection and Treatment of Active Compounds and Targets in QSP

The chemical components of CS, BC, CR, STR, and HMM meeting the criteria of oral bioavailability (OB) >30% and drug likeness (DL) >0.18 were selected as candidate active components using the Traditional Chinese Medicine Systems Pharmacology (TCMSP) database [11], and their potential targets were retrieved [12, 13]. The chemical components of MO were determined using the BATMAN-TCM server [14], and the principle of “drug-target similarity” was used to set the threshold “score cutoff = 30” to calculate the potential targets corresponding to chemical components.

### 2.2. Prediction of the Targets of Active Ingredients and Construction of the Active Ingredient-Target Network Map of QSP

Potential protein targets of QSP were screened using the TCMSP database, and all retrieved target proteins were corrected to the official symbols using the UniProt database. The network diagram of “active component-biological target” was constructed using Cytoscape 3.2.1 software [15]. Nodes in the network represent active components and biological targets. Active components and biological targets were connected by edges.

### 2.3. Retrieval of Relevant Biological Targets for NP

In this study, “neuropathic pain” was used as the search term to retrieve recognized biological targets using various databases (TTD [16], DrugBank [17], DisGeNET [18], GAD [19], and PharmGKB [20]), and the search results were used to identify currently known biological targets related to NP. Because of the poor uniformity of biological target information sources, the UniProt ID of each target was obtained by correcting the target protein and gene information screened via protein database retrieval [21].

### 2.4. Construction of the Protein-Protein Interaction (PPI) Network Diagram and Screening of Key Biological Targets

The network diagram of PPIs is an important method for identifying direct and indirect interactions between targets [22]. The PPI network was constructed using the STRING platform, and the species was set as “*Homo sapiens* [23].” The minimum threshold of interaction was set as “medium confidence = 0.4,” which was used to draw the PPI maps of the active components of QSP and PPI maps of NP-related targets. The “Network Analyzer” function of Cytoscape 3.2.1 [24] was utilized to analyze the topological properties of PPI network and calculate two important topological parameters: “node degree distribution” and “betweenness centrality” of the overall network. The node degree reflects the number of connections with other nodes, whereas betweenness reflects a network of all of the shortest paths through a node in the path. These measures indicate the importance of a node in the network and determine whether a target protein is a “core target.” To study the possible pharmacological effects of a core target, the mean degree and betweenness are taken as the “card value,” and the target between degree and betweenness on the card value is selected as the “core target [25].”

### 2.5. Gene Ontology (GO) Enrichment Analysis of NP-Related Targets following QSP Treatment

To illustrate the QSP treatment target NP protein in the role of gene function, this study uses David [26] analysis QSP PPI for the treatment of NP protein involved in the network GO function analysis of enrichment. According to the number of targets involves sorting, the first few biological processes establish GO bioprocess enrichment and analyze the possible bioprocesses of core targets in the body.

### 2.6. Kyoto Gene and Genomics Encyclopedia (KEGG) Pathway Enrichment Analysis of the Targets of QSP in the Treatment of NP

Using DAVID, the protein targets of QSP in the treatment of NP were examined using KEGG signaling pathway enrichment [27]. The correlation between core targets and NP in the KEGG signal pathway and clear QSP possible pharmacological mechanism is researched. By using Cytoscape 3.2.1 software, the KEGG pathway of “drug—active ingredient—target—pathway” network is constructed. The results of the enrichment analysis of visualization were processed by ImageGP software.

## 3. Results

### 3.1. Screening of the Active Ingredients in QSP

QSP consists of CS, BC, CR, STR, HMM, and MO. Using the TCMSP database, a total of 361 compounds were retrieved, including 16 in CS, 19 in BC, 189 in CR, 50 in STR, and 87 in HMM. Of the 16 compounds in CS, six satisfied the criteria of OB ≥ 30% and DL ≥ 0.18. Of the 19 compounds in BC, five satisfied the criteria of OB ≥ 30% and DL ≥ 0.18. Of the 189 compounds in CR, 115 satisfied the criteria of OB ≥ 30%, whereas seven satisfied the criteria of OB ≥ 30% and DL ≥ 0.18. Of the 50 compounds in STR, all satisfied the criteria of OB ≥ 30% and four satisfied the criteria of OB ≥ 30% and DL ≥ 0.18. Of the 87 compounds in HMM, 44 satisfied the criteria of OB ≥ 30% and 20 satisfied the criteria of OB ≥ 30% and DL ≥ 0.15. Thus, 42 of the total 361 compounds satisfied the criteria, and 37 compounds were finally obtained after excluding duplicates. BATMAN-TCM was used to excavate 30 active components of MO, and 25 active components were obtained after excluding five components not meeting the score cutoff of 30. The remaining 60 active ingredients were removed after deleting duplicates. The resulting network of the final selected compounds from the six herbal medicines is shown in Table 1.

### 3.2. Prediction of the Targets of QSP and Construction of an Active Component-Target Network Diagram

**Figure 2:**
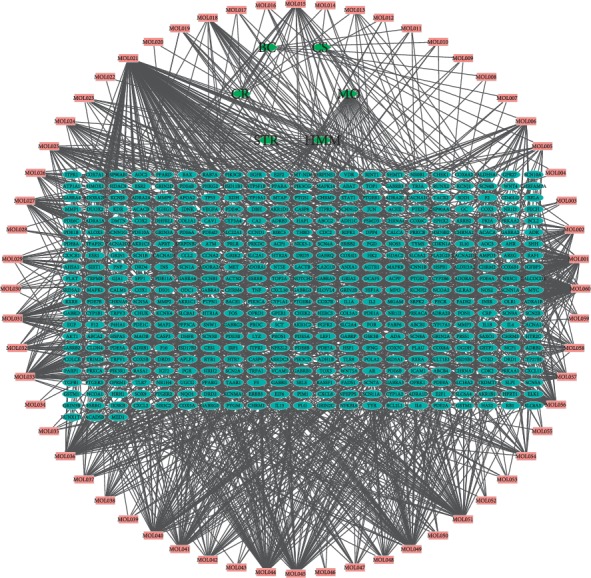
The herb-compound-target gene network consisting of six herbal medicines (green hexagons), 60 compounds (pink rectangles), and 444 target genes (sky-blue ovals).

Using the TCMSP database, 706 potential targets of CS, BC, CR, STR, and HMM were screened, and 734 potential targets of MO in astragalus musk deer pills were screened using the BATMAN-TCM database. The remaining 444 targets were eliminated after deleting duplicates. Compounds were linked to biological targets, and networks of active-component-target interactions were plotted using Cytoscape 3.2.1 software. Network diagrams more intuitively reflect the interactions of these compounds with targets. It was found that the network diagram contained 504 nodes, and the interaction network between drugs and targets contained 1425 edges. The 504 nodes included 60 active components of QSP. Quercetin acted on 151 targets, and beta-sitosterol acted on 74 targets. PTGS2 had a total of 28 potential targets, followed by 24 targets for PTGS1. This fully reflects the complex network relationship between multiple targets, verifying that QSP functions in a multichannel, multilink, multitarget, and integrated synergistic manner. These genes, proteins, compounds, and herbs are listed in Supplementary Table 1, and the resulting herb-compound-target gene network is shown in Figure 2. The key nodes and topological properties of the active component-target network are shown in Table 2.

### 3.3. Retrieval of NP-Related Targets

By searching databases such as TTD, DrugBank, DisGeNET, GAD, and PharmGKB, a total of 70 QSP targets related to the occurrence and development of NP were identified after deleting repeated targets, as shown in Supplementary Table 2.

### 3.4. Construction of the PPI Network for the Treatment of NP by the Active Ingredients of QSP and the Screening and Analysis of Key Targets

**Figure 3:**
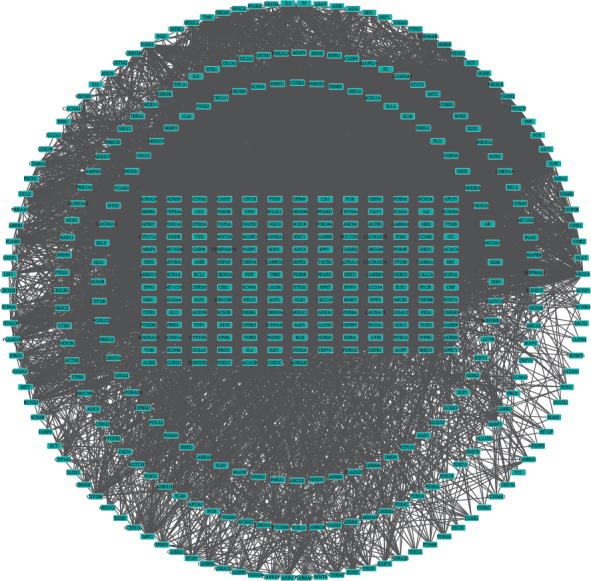
The PPI network of the active components of QSP.

**Figure 4:**
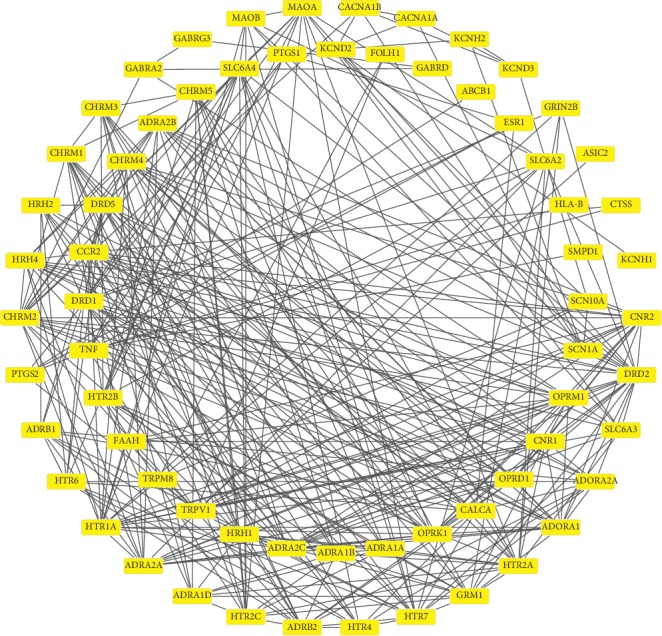
The PPI network graph of NP-related targets.

**Figure 5:**
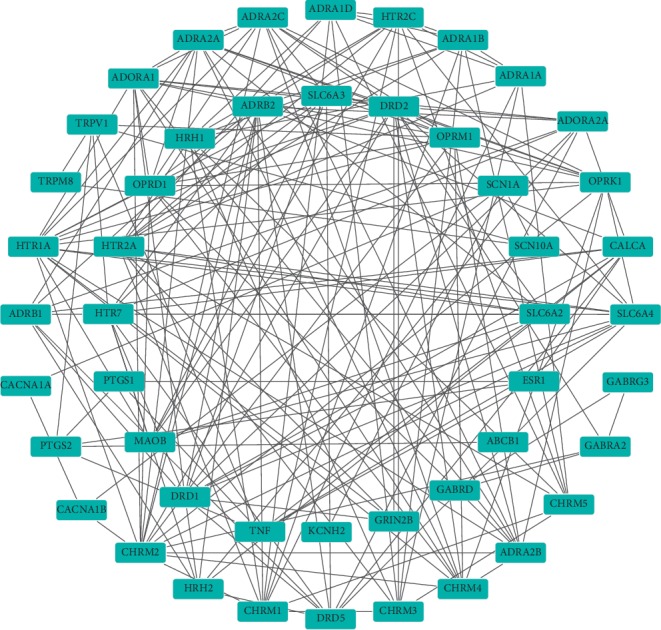
The PPI network of the NP-related targets of the active ingredients of QSP.

The PPI network of the active components of QSP was constructed using STRING (Figure 3), and the results revealed 434 direct or indirect targets and 7070 interactions between the targets. At the same time, the PPI network graph of NP-related targets identified 66 targets directly or indirectly related to the selected targets (Figure 4). In total, 324 interactions were identified between the targets. Cytoscape was used to extract the intersection network in the PPI network graph and delete targets without intersections. The screening principle was that when the active components in the vine acted on an NP target, the active components were associated with NP. In total, 48 potential targets were analyzed. There were 191 interactions between targets.  Figure 5 presents the PPI network of the NP-related targets of the active ingredients of QSP.  Table 3 presents information about the targets in the PPI network in the treatment of NP by QSP. Cytoscape 3.2.1 software was used to create an active component-target interaction network. The network map more intuitively reflects the interactions of the active components of QSP with NP-related targets.

**Figure 6:**
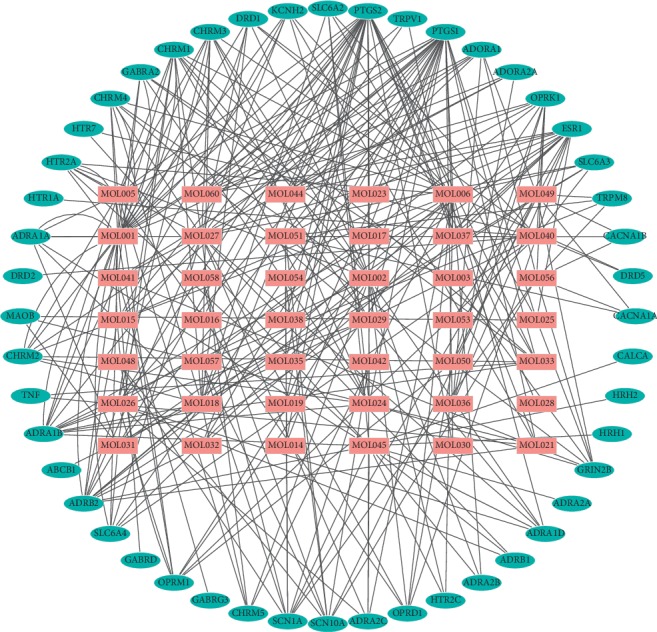
The active ingredients of QSP and NP target network through a network of 42 compounds (pink rectangles) and 48 target genes (sky-blue ovals).

Analysis of the network of active ingredient-target interactions revealed that the network map contained 90 nodes and 296 edges. The 90 nodes included 42 active components of QSP in blood and 48 targets of QSP. The active ingredients of QSP and NP target network are shown in Figure 6. In the subsequent experiment, Network Analyzer topology properties were used to filter key targets. After a target is screened according to the network topology theory, it can affect more nodes, increasing the efficiency of network information transmission. This study screened 10 key targets: DRD2, CHRM2, *β*2-adrenergic receptor (ADRB2), HTR7, OPRM1, HTR2A, SLC6A4, HTR2C, CHRM1, and calcitonin gene-related peptide (CALCA). The interaction between the target and the target is 18 sides.  Table 4 shows the 10 key nodes and the topological properties of QSP in the NP-related target PPI network.

### 3.5. GO Biological Process Annotation for NP-Related Targets of QSP

Using the GO bioprocess enrichment analysis function of the DAVID platform, the functions of the 48 proteins in the PPI network of QSP in the treatment of NP were studied, and 53 GO entries were determined using a false discovery rate (FDR) of <0.05. The 53 GO entries are listed in Supplementary Table 3. GO is an analytical method for classifying genes according to their function, which can be divided into three parts: biological process, molecular function, and cell composition. GO analysis identifies important functions that cause a trait to change, and the gene corresponding to the function is revealed. Among them, 35 items were related to biological processes, including the regulation of systemic processes, multicellular biological processes, signal transduction, cell proliferation, and response to stimuli. Meanwhile, seven items related to molecular function were found, including items linked to receptor activity, neurotransmitter binding, drug binding, and adrenaline binding, and 11 related items were related to cell composition, including items related to the plasma membrane, synapses, and dendrites.

### 3.6. KEGG Pathway Enrichment Analysis of QSP in the Treatment of NP

**Figure 7:**
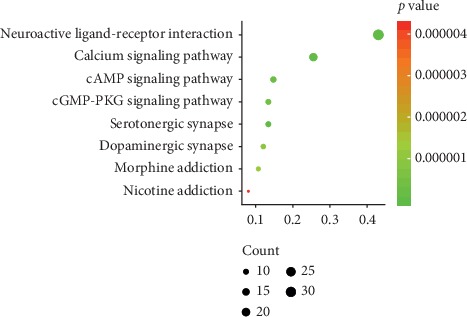
The bubble diagram of KEGG mechanism analysis.

KEGG pathway enrichment analysis was performed using DAVID. The pathways of 48 proteins involved in PPI network of NP-related targets of QSP were analyzed via pathway enrichment, and 24 signaling pathways were obtained. According to the principle of FDR <0.05, eight signaling pathways, including calcium signaling and neural active ligand-receptor interaction signaling pathways, were identified, indicating that the active components of QSP can act on these signaling pathways in the treatment of NP.  Table 5 shows the pathways for QSP in the treatment of NP target. The enrichment analysis results were visualized using Image GP software. The bubble size in the figure represents the number of genes enriched in the pathway. The difference in bubble color represents the degree of enrichment of the target gene in the pathway. The bubble diagram is shown in Figure 7. In this study, 32 genes were enriched in the neuroactive ligand-receptor interaction signaling pathway, 19 genes were enriched in the calcium signaling pathway, and 11 genes were enriched in the cAMP signaling pathway.

### 3.7. Construction of the Drug-Active Ingredient-Target-Pathway Network Diagram of QSP in the Treatment of NP

**Figure 8:**
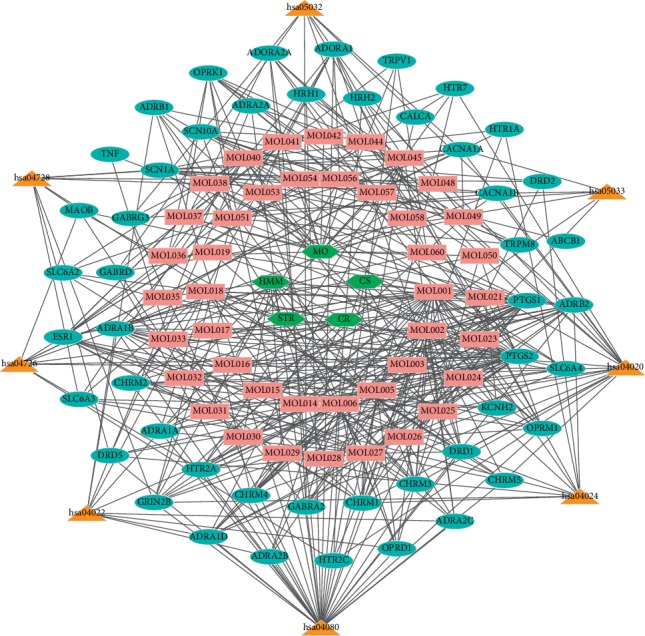
The drug-active ingredient-target-pathway network diagram of a network of five herbs (green hexagons), 42 compounds (pink rectangles), 48 target genes (sky-blue ovals), and 8 pathways (orange triangles).

The drug-active ingredient-target-pathway network model map of QSP was constructed using Cytoscape 3.2.1 software. The map included 103 nodes, including 5 drugs, 42 active components, and 48 target proteins. The metabolic pathways were connected by 444 edges to form a complete network.  Figure 8 presents the drug-active ingredient-target-pathway network diagram.

## 4. Discussion

TCMSP is a system-level pharmacological database of TCM that provides systematic TCM information, and it is a platform for the development of new drugs for the treatment of human diseases. After a critical review of pharmacological and clinical knowledge, the TCMSP collected all 499 herbs registered in the 2010 edition of the Chinese Pharmacopoeia, totaling 12,144 compounds [11]. BATMAN-TCM is the first online bioinformatics analysis tool of TCM molecular mechanisms, and it is used to clarify the “multicomponent, multitarget, and multipathway” combinatorial therapeutic mechanism of TCM and provide valuable clues for subsequent experimental validation, thus accelerating the elucidation of the molecular mechanisms of TCMs [14]. It is the first step of network pharmacology analysis for identifying chemical components and their target information as completely as possible. The collection of the chemical constituents of QSP is accurate and complete using the two databases currently recognized for network pharmacology research.

QSP can relieve pain and promote blood circulation. The drug has been widely used in China. Clinical studies revealed that QSP can significantly improve mild-to-moderate NP and improve the quality of life of patients with mild-to-moderate NP, especially concerning physiological function, social function, emotional function, vitality, and other aspects. In a number of randomized, double-blind, placebo-controlled studies, improvements in dysfunction associated with mild-to-moderate NP were observed in both the QSP and placebo group, although the improvement was significantly better in the QSP group [5]. To study the mechanism of action of QSP, this study relied on the TCMSP and BATMAN-TCM platforms to study the active constituents of six Chinese herbs (CS, BC, CR, STR, HMM, and MO) and constructed a compound-target network to analyze the direct interactions between compounds and targets. These findings provided a reference for the multicomponent, multitarget, and multipath treatment mechanism of QSP. The results of this study suggest that QSP most likely exerts its therapeutic effects on NP through interactions among MOL021, MOL001, PTGS2, and PTGS1, thereby laying a foundation for subsequent confirmatory studies. The key compounds of QSP in the target network included quercetin and beta-sitosterol, which had much higher degrees than other compounds, and beta-sitosterol was detected in both CS and STR. It can thus be speculated that CS and STR play key roles in the effects of QSP [28]. Prior research indicated that beta-sitosterol has a wide range of pharmacological effects including anti-inflammatory, immunomodulatory, antioxidant, and analgesic effects [29]. Quercetin can lower blood pressure, enhance capillary resistance, and reduce capillary fragility [30]. The key targets of QSP in the key target network included PTGS1 and PTGS2. PTGS is a key rate-limiting enzyme for the synthesis of prostaglandins from arachidonic acid [31], and two isomerases, namely, PTGS1 and PTGS2, have been identified. PTGS and its products are involved in various physiological and pathological processes such as inflammatory responses and blood coagulation balance [32]. Ten key target proteins were screened using the Network Analyzer topology properties in the PPI network for the treatment of NP by the active ingredients of QSP. It was previously found that DRD2 is the major receptor for NP [33], and KLF15 is induced in NP in a TNF-α-dependent manner. KLF15 induces NP by promoting the expression of the DRD2 receptor. NP is associated with persistent changes in gene expression in primary sensory neurons, but the underlying epigenetic mechanisms leading to these changes are unclear. In addition, CHRM plays a key role in the regulation of spinal cord nociceptive transmission [34]. Norepinephrine and the adrenergic system control the transmission of damage at the level of the spinal cord and play a key role in this process. Stimulation of ADRB2 downregulates the phosphorylation of p38 MAPK in microglia and JNK in astrocytes. The aforementioned studies indicated that spinal ADRB2 regulates MAPK phosphorylation and downregulates glial activity, thereby inhibiting neuropathic damage conduction at the spinal cord level [35]. The HTR family is involved in the central nociceptive mechanism, which plays a key role in suppressing analgesic pathways, and its stimulation can regulate neuronal signaling induced by neuropathy [36]. Animal studies revealed that CALCA expressed in dorsal root ganglia is involved in the generation and maintenance of NP [37]. Based on the aforementioned information, it is speculated that the testes directly or indirectly act on these key targets to play a pharmacological role in the treatment of NP.

PPI networks are composed of individual proteins interacting with each other to participate in biological signal transmission, gene expression regulation, energy and substance metabolism, cell cycle regulation, and other aspects of life processes. It is of great significance to understand the functional connection between proteins [22]. Enrichment analysis is helpful for studying gene and expression information as a whole network. In organisms, different gene products coordinate with each other to perform biological functions, and pathway annotation analysis of differentially expressed genes is helpful for further interpreting gene functions [26, 27]. In this study, we preliminarily explored the primary pathway of QSP in treating NP using PPI network and enrichment analyses. In the present study, the analysis yielded the following results. Of the 5 herbs and 42 compounds examined, a total of 48 target genes were associated with NP. To illustrate the role of the active targets of QSP in gene function and signaling pathways, GO functional enrichment and KEGG pathway enrichment analyses of the 48 genes in the PPI network were performed. GO function enrichment analysis found that the effects of QSP in the treatment of NP are mainly reflected in the regulation of receptor activity, plasma membrane, and systemic processes. The main KEGG pathways involved in the treatment of NP by QSP included calcium signaling and neuroactive ligand-receptor interaction signaling pathways. From the aforementioned studies, it is concluded that the active components of QSP alleviate NP by acting on these signaling pathways. A neuroactive ligand-receptor interaction signaling pathway is a collection of receptor ligands on the plasma membrane that are associated with intracellular and extracellular signaling pathways and are closely related physiologically to neurological function [38]. Key molecules in the calcium signaling pathway have a variety of biological functions, among which calcium ions play an important role in maintaining biopotential [39], maintaining normal nerve conduction function [40], and regulating physiological actions including intracellular enzyme activity, muscle contraction and relaxation [41], nervous system function [42], stimulation and secretion-coupled participation [43], glucose metabolism [44], and prostaglandin [45] and insulin synthesis [46].

Network pharmacology combines the concepts of systems biology and multidirectional pharmacology [47]. It explores the relationship between drugs and diseases from a holistic perspective, emphasizing the holistic and systematic interactions among drugs, targets, and diseases [48]. The Chinese herb system is a multicomponent complex system. Bioinformatic and network pharmacology research helps to both clarify the mechanism of action of QSP in the treatment of NP and provide an important theoretical basis for further discussion of subsequent molecular mechanisms and optimization of experimental design. This research also provides new ideas and methods for the research and development of new Chinese herbs and elucidation of the related mechanisms of drugs and diseases [49]. Meanwhile, some limitations of this study must be addressed. First, because of limitations regarding screening conditions, only the main compounds in QSP could be analyzed, and thus the research results are limited to some extent. Second, although a large number of targets and pathways can be screened using network pharmacology, these results need to be verified via pharmacological experiments. Third, further validation experiments are needed to increase the drug concentration to accurately reveal the effective ingredients and mechanism of QSP in the treatment of NP.

## 5. Conclusion

The network pharmacology analysis of QSP identified 444 compounds in six herbs, and 48 of the compounds in the six herbs were screened to identify NP-related target genes. This study demonstrated that network pharmacology studies can reveal close interactions between multiple components and multiple targets. The network pharmacology approach of this study provides another research strategy for a comprehensive understanding of the mechanism by which QSP treats NP.

## Figures and Tables

**Table 1:** The resulting network of the final selected compounds from the six herbal medicines.

Number	Molecule name	OB	DL	Herb name
MOL001	Beta-sitosterol	36.91	0.75	CS/STR
MOL002	16-Epi-isositsirikine	49.52	0.59	CS
MOL003	Magnograndiolide	63.71	0.19	CS
MOL004	Michelenolide	47.54	0.25	CS
MOL005	Sinomenine	46.09	0.53	CS
MOL006	Stepholidine	33.11	0.54	CS
MOL007	CLR	37.87	0.68	BC
MOL008	Methyl (4R)-4-[(3R,5S,7S,8R,9S,10S,12S,13R,14S,17R)-3,7,12-trihydroxy-10,13-dimethyl-2,3,4,5,6,7,8,9,11,12,14,15,16,17-tetradecahydro-1H-cyclopenta[a]phenanthren-17-yl]pentanoate	32.32	0.76	BC
MOL009	Methyl desoxycholate	34.63	0.73	BC
MOL010	Deoxycholic acid	40.72	0.68	BC
MOL011	ZINC01280365	46.38	0.49	BC
MOL012	Sitosterol	36.91	0.75	CR
MOL013	FA	68.96	0.71	CR/HMM
MOL014	Mandenol	42	0.19	CR
MOL015	Myricanone	40.6	0.51	CR
MOL016	Perlolyrine	65.95	0.27	CR
MOL017	Wallichilide	42.31	0.71	CR
MOL018	*N*-Methylflindersine	32.36	0.18	STR
MOL019	Hesperetin	70.31	0.27	STR
MOL020	(3S,8S,9S,10R,13R,14S,17R)-10,13-Dimethyl-17-[(2R,5S)-5-propan-2-yloctan-2-yl]-2,3,4,7,8,9,11,12,14,15,16,17-dodecahydro-1H-cyclopenta[a]phenanthren-3-ol	36.23	0.78	HMM
MOL021	Quercetin	46.43	0.28	HMM
MOL022	Mairin	55.38	0.78	HMM
MOL023	Jaranol	50.83	0.29	HMM
MOL024	Hederagenin	36.91	0.75	HMM
MOL025	Isorhamnetin	49.6	0.31	HMM
MOL026	3,9-Di-O-methylnissolin	53.74	0.48	HMM
MOL027	7-O-Methylisomucronulatol	74.69	0.3	HMM
MOL028	9,10-Dimethoxypterocarpan-3-O-*β*-D-glucoside	36.74	0.92	HMM
MOL029	(6aR,11aR)-9,10-Dimethoxy-6a,11a-dihydro-6H-benzofurano [3,2-c]chromen-3-ol	64.26	0.42	HMM
MOL030	Bifendate	31.1	0.67	HMM
MOL031	Formononetin	69.67	0.21	HMM
MOL032	Calycosin	47.75	0.24	HMM
MOL033	Kaempferol	41.88	0.24	HMM
MOL034	Isomucronulatol-7,2′-di-O-glucosiole	49.28	0.62	HMM
MOL035	1,7-Dihydroxy-3,9-dimethoxy pterocarpene	39.05	0.48	HMM
MOL036	Androst-4-ene-3,17-dione	—	—	MO
MOL037	Testosterone	—	—	MO
MOL038	3,5-Dihydroxybenzoic acid	—	—	MO
MOL039	Musennin	—	—	MO
MOL040	17-Beta-estradiol	—	—	MO
MOL041	Allantoin	—	—	MO
MOL042	3-Methylcyclotridecan-1-one	—	—	MO
MOL043	Muscopyridine	—	—	MO
MOL044	5-*cis*-Cyclopentadecen-1-one	—	—	MO
MOL045	Decamine	—	—	MO
MOL046	Musclide A1	—	—	MO
MOL047	Estragole	—	—	MO
MOL048	Cyclotetradecan-1-one	—	—	MO
MOL049	3beta-Hydroxy-5alpha-androstan-17-one	—	—	MO
MOL050	Alpha-estradiol	—	—	MO
MOL051	Androsterone	—	—	MO
MOL052	Cyclovirobuxine	—	—	MO
MOL053	Cholesterol	—	—	MO
MOL054	Muscone	—	—	MO
MOL055	2,6-Decamethylene pyridine	—	—	MO
MOL056	3alpha-Hydroxy-5alpha-androstan-17-one	—	—	MO
MOL057	Muscol	—	—	MO
MOL058	Normuscone	—	—	MO
MOL059	2,6-Nonamethylene pyridine	—	—	MO
MOL060	5-*cis*-Cyclotetradecen-1-one	—	—	MO

**Table 2:** The key nodes and topological properties of the active component-target network.

Name	Category	Degree	Betweenness
MOL021	Compound	152	0.36805645
MOL001	Compound	76	0.0386247
PTGS2	Target	28	0.04871355
PTGS1	Target	24	0.04082273

**Table 3:** The gene, target name, and Uniprot ID in the PPI network in the treatment of NP by QSP.

Number	Gene	Target name	Uniprot ID
1	ABCB1	Multidrug resistance protein 1	P08183
2	ADORA1	Adenosine receptor A1	P30542
3	ADORA2A	Adenosine receptor A2a	P29274
4	ADRA1A	Alpha-1A adrenergic receptor	P35348
5	ADRA1B	Alpha-1B adrenergic receptor	P35368
6	ADRA1D	Alpha-1D adrenergic receptor	P25100
7	ADRA2A	Alpha-2A adrenergic receptor	P08913
8	ADRA2B	Alpha-2B adrenergic receptor	P18089
9	ADRA2C	Alpha-2C adrenergic receptor	P18825
10	ADRB1	Beta-1 adrenergic receptor	P08588
11	ADRB2	Beta-2 adrenergic receptor	P07550
12	CACNA1A	Voltage-dependent P/Q-type calcium channel subunit alpha-1A	O00555
13	CACNA1B	Voltage-dependent *N*-type calcium channel subunit alpha-1B	Q00975
14	CALCA	Calcitonin gene-related peptide 1	P06881
15	CHRM1	Muscarinic acetylcholine receptor M1	P11229
16	CHRM2	Muscarinic acetylcholine receptor M2	P08172
17	CHRM3	Muscarinic acetylcholine receptor M3	P20309
18	CHRM4	Muscarinic acetylcholine receptor M4	P08173
19	CHRM5	Muscarinic acetylcholine receptor M5	P08912
20	DRD1	Dopamine D1 receptor	P21728
21	DRD2	D(2) dopamine receptor	P14416
22	DRD5	D(1B) dopamine receptor	P21918
23	ESR1	Estrogen receptor	P03372
24	GABRA2	Gamma-aminobutyric-acid receptor alpha-2 subunit	P47869
25	GABRD	Gamma-aminobutyric acid receptor subunit delta	O14764
26	GABRG3	Gamma-aminobutyric acid receptor subunit gamma-3	Q99928
27	GRIN2B	Glutamate ionotropic receptor NMDA type subunit 2B	Q13224
28	HRH1	Histamine H1 receptor	P35367
29	HRH2	Histamine H2 receptor	P25021
30	HTR1A	5-Hydroxytryptamine receptor 1A	P08908
31	HTR2A	5-Hydroxytryptamine 2A receptor	P28223
32	HTR2C	5-Hydroxytryptamine 2C receptor	P28335
33	HTR7	5-Hydroxytryptamine receptor 7	P34969
34	KCNH2	Potassium voltage-gated channel subfamily D member 2	Q9NZV8
35	MAOB	Amine oxidase [flavin-containing] B	P27338
36	OPRD1	Delta-type opioid receptor	P41143
37	OPRK1	Opioid receptor kappa 1	P41145
38	OPRM1	Opioid receptor mu 1	P35372
39	PTGS1	Prostaglandin G/H synthase 1	P23219
40	PTGS2	Prostaglandin G/H synthase 2	P35354
41	SCN10A	Sodium voltage-gated channel alpha subunit 10	Q9Y5Y9
42	SCN1A	Sodium voltage-gated channel alpha subunit 1	P35498
43	SLC6A2	Sodium-dependent noradrenaline transporter	P23975
44	SLC6A3	Sodium-dependent dopamine transporter	Q01959
45	SLC6A4	Sodium-dependent serotonin transporter	P31645
46	TNF	Tumor necrosis factor	P01375
47	TRPM8	Transient receptor potential cation channel subfamily M member 8	Q7Z2W7
48	TRPV1	Transient receptor potential cation channel subfamily V member 1	Q8NER1

**Table 4:** Ten key nodes in QSP treatment of NP target PPI networks and their topological properties.

Name	Degree	Betweenness
DRD2	17	0.0853782
CHRM2	15	0.2364376
ADRB2	12	0.10820742
HTR7	12	0.07132715
OPRM1	12	0.06858521
HTR2A	12	0.06496394
SLC6A4	11	0.12727011
HTR2C	11	0.04633399
CHRM1	10	0.08797099
CALCA	9	0.04503978

**Table 5:** The pathways for QSP in the treatment of NP target (FDR < 0.05).

Name	Term	Count	Genes	*P* value	FDR
hsa04080	Neuroactive ligand-receptor interaction	32	OPRM1, DRD1, TRPV1, ADORA2A, DRD2, DRD5, OPRK1, ADORA1, HRH1, HTR1A, GRIN2B, HRH2, ADRA2A, ADRA2C, ADRA2B, GABRD, GABRA2, GABRG3, ADRB2, CHRM5, ADRB1, CHRM4, CHRM3, CHRM2, HTR7, CHRM1, ADRA1B, ADRA1A, HTR2C, ADRA1D, HTR2A, OPRD1	1.05*E* − 34	1.18*E* − 31

hsa04020	Calcium signaling pathway	19	DRD1, ADORA2A, DRD5, CHRM5, HRH1, ADRB2, ADRB1, CHRM3, HRH2, CHRM2, CHRM1, HTR7, ADRA1B, ADRA1A, HTR2C, ADRA1D, CACNA1A, HTR2A, CACNA1B	4.24*E* − 18	4.77*E* − 15

hsa04726	Serotonergic synapse	10	HTR1A, PTGS2, HTR7, SLC6A4, PTGS1, MAOB, HTR2C, CACNA1A, HTR2A, CACNA1B	1.91*E* − 08	2.14*E* − 05

hsa04024	cAMP signaling pathway	11	DRD1, ADRB2, HTR1A, ADRB1, GRIN2B, ADORA2A, DRD2, CHRM2, DRD5, CHRM1, ADORA1	2.61*E* − 07	2.93*E* − 04

hsa04022	cGMP-PKG signaling pathway	10	ADRB2, ADRB1, ADRA2A, ADRA1B, ADRA1A, ADRA2C, ADRA2B, ADORA1, ADRA1D, OPRD1	4.09*E* − 07	4.60*E* − 04

hsa04728	Dopaminergic synapse	9	DRD1, SCN1A, GRIN2B, DRD2, SLC6A3, DRD5, MAOB, CACNA1A, CACNA1B	9.69*E* − 07	0.001089

hsa05032	Morphine addiction	8	OPRM1, GABRD, DRD1, GABRA2, GABRG3, ADORA1, CACNA1A, CACNA1B	1.23*E* − 06	0.001381

hsa05033	Nicotine addiction	6	GABRD, GABRA2, GABRG3, GRIN2B, CACNA1A, CACNA1B	4.20*E* − 06	0.004726

## Data Availability

The data used to support the findings of this study are available from the corresponding author upon request.

## Supplementary Materials

Supplementary Materials Supplementary Table 1: list of the genes, proteins, compounds, and herbs of QSP. Supplementary Table 2: list of a total of 70 QSP targets related to the occurrence and development of NP. Supplementary Table 3: list of the 53 GO entries.
